# CD44 Expression in Intestinal Epithelium and Colorectal Cancer Is Independent of p53 Status

**DOI:** 10.1371/journal.pone.0072849

**Published:** 2013-08-29

**Authors:** Jurrit Zeilstra, Sander P. J. Joosten, Louis Vermeulen, Jan Koster, Jan Paul Medema, Rogier Versteeg, Marcel Spaargaren, Steven T. Pals

**Affiliations:** 1 Department of Pathology, Academic Medical Center, University of Amsterdam, Amsterdam, The Netherlands; 2 Laboratory for Experimental Oncology and Radiobiology (LEXOR), Center for Experimental Molecular Medicine, Academic Medical Center, University of Amsterdam, Amsterdam, The Netherlands; 3 Department of Oncogenomics, Academic Medical Center, University of Amsterdam, Amsterdam, The Netherlands; National Cancer Centre, Singapore

## Abstract

CD44 marks stem cell-like cells in a number of tumour types, including colorectal cancer (CRC), while aberrant CD44 expression conveys increased tumourigenic, invasive, and metastatic potential. Previous data indicate that CD44 is a direct target of p53-mediated transcriptional repression in breast cancer. Since inactivating *p53* mutations are frequent genetic events in CRC these could unleash expression of CD44. In the present study, we therefore explored the relation between *p53* mutational status and CD44 expression in a cohort of 90 localized primary CRCs and studied the effect of radiation-induced p53 activation on CD44 expression. Interestingly, we observed that, in contrast to breast cancer, loss of function *p53* mutations were not associated with elevated CD44 expression in colon cancer. Moreover, DNA-damage induced p53 activation did not result in repression of CD44 expression, neither in colon cancer cells nor in normal intestinal epithelial cells. Our data demonstrate that CD44 expression in normal and malignant intestinal epithelial cells is not regulated by p53, implying that regulation of this potentially important therapeutic target is tissue and cancer-type specific.

## Introduction

CD44 comprises a family of cell adhesion and signalling molecules that exert pleiotropic effects on important biological processes including proliferation, survival, migration, epithelial to mesenchymal transition (EMT), and cancer metastasis (reviewed by Zöller [1]). In the intestinal mucosa, CD44 is a major direct target of Wnt signalling and is prominently expressed on intestinal stem cells [2]–[4]. There is accumulating evidence that CD44 is involved in the initiation and progression of intestinal tumours and the development of metastasis [1], [3], [5]–[9]. In addition, prominent expression of CD44 is a hallmark of highly tumourigenic CRC cells [10]. Accordingly, it was recently demonstrated that *CD44* is part of an intestinal stem cell gene signature that predicts disease relapse in CRC patients [11]. This signature was specifically associated with CRC cells endowed with high-tumour initiating potential as well as long-term self-renewal capacity. Hence, CD44 represents a potential therapeutic target for the treatment of CRC and it is therefore important to understand the different mechanisms that underlie the regulation of CD44. In the majority of cases of CRC, expression of CD44 is increased as a result of dysregulated Wnt/β-catenin signalling [2], [12]. However, there is ample evidence that other not-yet identified pathways and mechanisms contribute to the regulation of Wnt/β-catenin target gene expression in intestinal tumours [13]. The tumour suppressor protein p53 is a transcription factor that plays a critical role in the suppression of cancer. In response to oncogenic stress, such as DNA damage, activated p53 protein binds to sequence-specific DNA sites, thereby regulating the transcription of a wide range of target genes involved in cell cycle control and survival signalling [14]. Mutational inactivation of the *p53* gene is a frequent genetic event in the progression of many types of human tumours, including breast cancer and colorectal cancer (CRC) [15]. It was recently demonstrated that p53 transcriptionally represses *CD44* expression in both normal and tumour-derived mammary epithelial cells by direct binding to the *CD44* promoter [16]. This p53-dependent regulation of CD44 was observed in both human and mouse mammary glands, indicating an evolutionary conserved function. Importantly, down-regulation of CD44 expression was found to be a prerequisite for p53-dependent growth regulation and induction of apoptosis in mammary epithelium [16]. A similar functional interplay between p53 and CD44 might also take place in intestinal epithelial cells and tumours. To explore whether CD44 expression is controlled by p53 protein in CRC, we analysed a cohort of primary colon carcinomas for *p53* mutational status and CD44 expression. Our study reveals that loss of p53 function is not associated with elevated CD44 expression in CRC. Furthermore, we demonstrate that activation of wild-type p53 is unable to repress CD44 expression in human colon cancer cells as well as in primary cultures of mouse intestinal crypt-villus organoids.

## Materials and Methods

### Ethical Statement

The study involving human biopsy samples was conducted in accordance with the Declaration of Helsinki and approved by the local ethics committee of The University of Amsterdam, AIEC (Algemene Instellingsgebonden Ethische Commissie). Patients gave written informed consent for the sample collection.

### Tumour Samples, p53 Mutation Analysis and Gene Expression Assay

The study cohort consisted of 90 AJCC stage II CRC patients that underwent intentionally curative surgery in the Academic Medical Center (AMC) in Amsterdam, The Netherlands, in the years 1997–2006 [17]. Representative fresh frozen tumour tissue was cut into 20 µm-thick sections that were immediately placed in TRIzol reagent (Invitrogen Life Technologies, Breda, the Netherlands), after which total RNA was extracted. Tumour load was examined routinely by an experienced pathologist. *p53* mutational status was determined using RT-PCR. In short, 2 µg of total RNA was reverse-transcribed in 25 µl reaction volume using pdN6 (Amersham Biosciences, Roosendaal, the Netherlands) and MMLV transcriptase (Gibco BRL, Breda, the Netherlands). PCR was performed on 1 µl of cDNA template using platinum Taq polymerase (Invitrogen Life Technologies). Oligo primers are listed in Table 1. PCR products were amplified by 35 cycles of 45 s at 95°C, 45 s at 60°C, and 1 min and 30 s at 72°C, and were sequenced directly using Big Dye Terminator Kit (Amersham) together with either sense or anti-sense oligo primer. Sequences were analysed using CodonCode Aligner software (CodonCode Corp., Dedham, MA). Gene expression levels in the tumours were assessed using the Affymetrix GeneChip Human Genome U133 Plus 2.0 array platform (Affymetrix, Santa Clara, CA). Purified RNA was processed, hybridized, and scanned according to the manufacturer’s protocol. Data was analysed using the software package R2 (http://r2.amc.nl), a web-based microarray analysis application developed by J.K. Data was MAS5-normalized and expression values were Log2 transformed. Statistical significance was assessed using one-way analysis of variance (ANOVA). Probe sets assayed were: *CDKN1A* (*p21*), ID: 202284_s_at; *MDM2,* 229711_s_at; and *CD44,* 209835_x_at. Other probe sets assaying *CD44* produced similar results, for example; 204489_s_at, *P*<0.01 and 210916_a_at, *P*<0.01).

**Table 1:** Oligo primers used for *p53* mutation analysis.

*Target*	*Orientation*	*Sequence (5′ to 3′)*
exon 1	sense	GCTTTCCACGACGGTGACA
exon 5	anti-sense	TTGTTGAGGGCAGGGGAGTA
exon 4	sense	TGTCATCTTCTGTCCCTTCC
exon 7	anti-sense	GATGGTGGTACAGTCAGAGC
exon 6	sense	TTGCGTGTGGAGTA
exon 11	anti-sense	GCAAGCAAGGGTTCAAAGACC

### Immunohistochemistry

Paraffin-embedded tumour tissue was stained using primary mAb mouse anti-human p53 (Dako, Glostrup, Denmark) and primary mAb mouse anti-human CD44 (VFF18) that recognizes CD44v6 [18]. Antibody binding was visualised using the Powervision poly-HRP detection system (ImmunoVision Technologies, Daly City, CA) and DAB+ (Dako). The intensity (I) of the staining was scored on semiquantitative scales as follows: “0”, no reaction; “1”, weak reaction; “2”, moderate reaction; and “3”, strong reaction. The extent of the signal was scored as percentage of positive cells (P). Overall staining score was calculated by multiplying the intensity by the percentage of positive cells (Score = P * I; maximum = 300). Fisher’s exact test was used for statistical analysis (*P*<0.001).

### Cell Culture, Immunoblotting and Real-time Reverse Transcription-PCR

RKO cells were cultured in McCoy’s 5A medium supplementented with 10% FCS until subconfluent. Mouse small intestinal crypts were isolated in accordance with protocols approved by the local animal ethics committee of The University of Amsterdam, DEC (Dier Ethische Commissie) and cultured for one week as described by *Sato et al*. [19]. Cultures were exposed to a single dose of 10 Gy from a ^137^Cs γ-ray radiation source at a dose rate of 0.8 Gy/min or incubated with 500 ng/mL neocarzinostatin (NCS) either in combination with 10 µM nutlin or not. Cells were harvested in lysis buffer at the indicated time points. Antibodies used for immunoblotting were anti-pan CD44 mAb Hermes-3 [20], anti-p21 mAb sx118 (Santa Cruz Biotechnology, Santa Cruz, CA), and anti-p53 mAb DO-1 (Santa Cruz Biotechnology). β-actin was used as loading control. In parallel, total RNA was isolated using the PicoPure RNA Isolation Kit (Arcturus Bioscience, Mountain View, CA) and real-time qRT-PCR was performed as described previously [3], [21]. One way-analysis of variance (ANOVA) was used to determine significant changes (*P*<0.05) in time.

## Results

### Loss of Function Mutation of p53 is Not Associated with Elevated CD44 Expression in Colon Cancer

**Figure 1:**
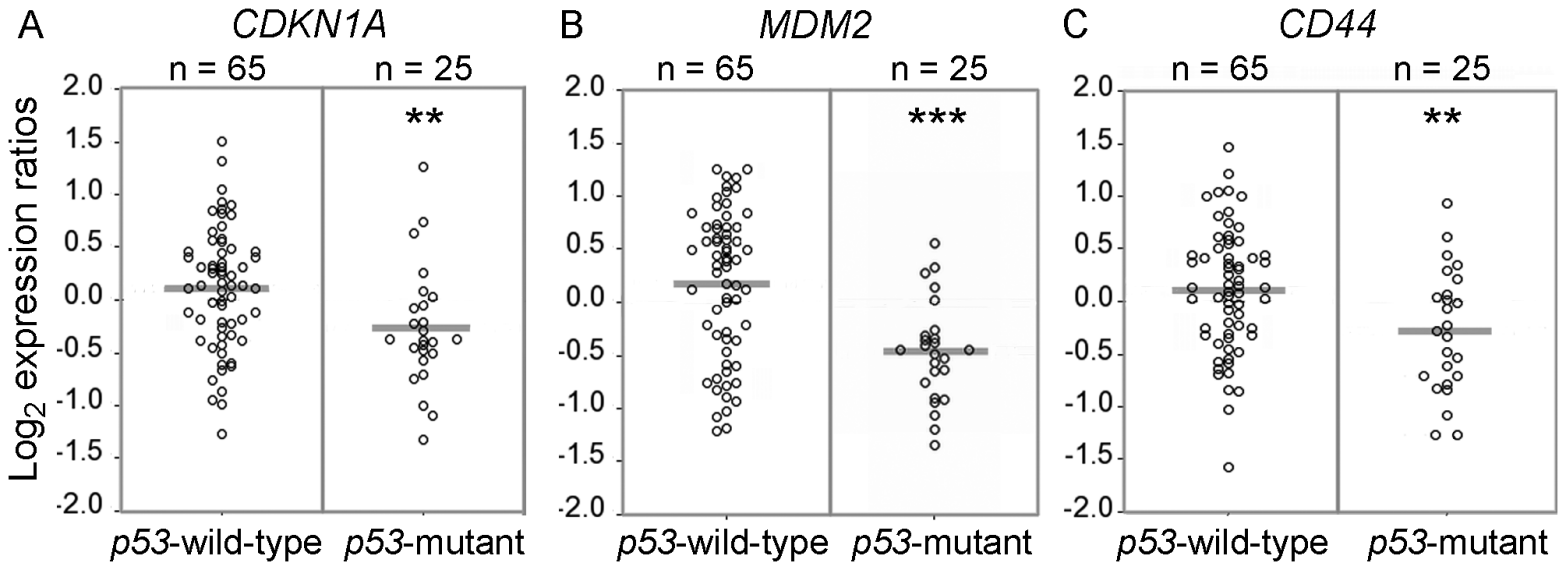
Loss of function mutation of *p53* is not associated with elevated *CD44* expression in colon cancer. Relative gene expression levels in *p53* mutant and *p53* wild-type adenocarcinomas for (A) *CDKN1A* (*p21*) (**, P<0.01), (B) *MDM2* (***, *P*<0.001), (C) *CD44,* (**, *P*<0.01).

The recent identification of p53 as a transcriptional repressor of *CD44* in breast cancer and mammary epithelium [16], prompted us to explore whether a similar functional relation exists in colon cancer and intestinal epithelium. We therefore examined the relation between p53 mutational status and *CD44* mRNA levels in a cohort of 90 colorectal carcinomas. All tumours included in this study were adenocarcinomas with invasion through the muscularis propria, but without lymph node or distant metastasis (Dukes B, AJCC Stage II). Mutational status was assessed by cDNA sequencing of the entire coding region of the *p53* gene, spanning exons 1 to 11. Sequence analysis identified 25 tumours (28%) with a mutation, resulting in a transcriptionally inactive p53 protein according to the definition of Soussi et al. [22] (Table 2). Comparison between the groups with wild-type and mutant *p53* revealed a significantly decreased mRNA expression of two canonical p53 transcriptional targets, *CDKN1A* (*p21*) (*P*<0.01) [23] and *MDM2* (*P*<0.001) [24] in the tumours with *p53* loss of function mutations (Figure 1A and B). Interestingly, in contrast to mammary tumours in which loss of p53 function was found to be significantly correlated with elevated *CD44* expression [16], *p53* mutation in colon carcinomas was correlated with decreased *CD44* mRNA expression levels (*P*<0.01; Figure 1C). These results imply that p53 does not act as a transcriptional repressor of *CD44* expression in CRC.

**Table 2:** *p53* mutations detected in colon adenocarcinomas.

*#*	*Sample*	*Gender*	*Age*	*Location*	*Mutation*	*Transactivation class (* * *)*
**1**	COL01	M	41	descending colon	Y205D	non-functional
**2**	COL06	M	76	transverse colon	R273P	non-functional
**3**	COL09	F	92	ascending colon	R273H	non-functional
**4**	COL10	F	54	cecum	P250L	non-functional
**5**	COL17	F	64	sigmoid colon	R273H	non-functional
**6**	COL35	M	67	cecum	R267W	non-functional
**7**	COL38	F	95	sigmoid colon	R282W	non-functional
**8**	COL44	M	78	transverse colon	R267P	non-functional
**9**	COL45	M	75	sigmoid colon	K132N	non-functional
**10**	COL48	F	90	hepatic flexure	T284P	non-functional
**11**	COL50	F	68	cecum	deletion Exon-9	non-functional
**12**	COL55	F	78	transverse colon	R175H	non-functional
**13**	COL59	F	34	cecum	F134C	non-functional
**14**	COL60	F	79	sigmoid colon	N235S & R249M	non-functional
**15**	COL61	M	78	cecum	deletion(AT) Codon 237	non-functional
**16**	COL62	F	80	sigmoid colon	R175H	non-functional
**17**	COL65	M	63	sigmoid colon	R175H	non-functional
**18**	COL68	M	74	sigmoid colon	R273H	non-functional
**19**	COL69	F	55	sigmoid colon	deletion(T) Codon 275	non-functional
**20**	COL73	M	80	descending colon	R175H	non-functional
**21**	COL74	M	74	sigmoid colon	C176Y	non-functional
**22**	COL76	M	69	sigmoid colon	R248Q	non-functional
**23**	COL79	M	72	ascending colon	R175H	non-functional
**24**	COL83	F	76	sigmoid colon	R342 Stop	non-functional
**25**	COL94	M	87	sigmoid colon	deletion Exon 7 Exon 8	non-functional

* p53 transactivation function according to Soussi T et al. [22].

### CD44 Protein Expression is not Increased in Colon Carcinomas with *p53* Mutation

**Figure 2:**
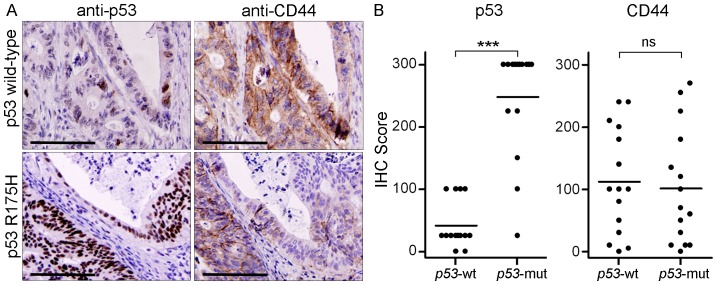
Loss of function mutation of *p53* is not associated with elevated CD44 protein expression in colon cancer. (A) Serial sections of a colon carcinoma without and with a p53 loss of function mutation (*i.e*., R175H, Table 2), stained for p53 and CD44 protein (bars indicate 50 µm). (B). Immunohistochemistry (IHC) score of p53 and CD44 protein expression, respectively (***, *P*<0.001, ns = not significant).

In order to confirm that *p53* mutational status and mRNA levels of *CD44* in primary colon cancer specimens reflect protein levels, we examined p53 and CD44 expression by immunohistochemistry in a subset of the tumours (n = 15/group). Mutations in *p53* often result in an inappropriate stabilization of the protein and nuclear accumulation [25]. In accordance, whereas tumours harbouring only wild-type *p53* gene sequences showed either no staining for p53 protein or nuclear staining in scattered cells, tumours containing a *p53* mutant gene showed a strong nuclear staining of the majority of the malignant cells (*P*<0.001, Figure 2A and B). CD44 expression was observed on the cell membrane of the vast majority tumours with either unmutated *p53* (14 out of 15) or mutated *p53* (14 out of 15) (Fig. 2A). Importantly, there was no significant difference in the CD44 staining score between tumours of both groups (*P*>0.05; Figure 2B). These findings demonstrate that, other than in breast cancer, loss of p53 function in colon cancer is not connected with increased CD44 protein expression.

### p53 does not Repress *CD44* in Colon Cancer Cells and Normal Intestinal Epithelium

**Figure 3:**
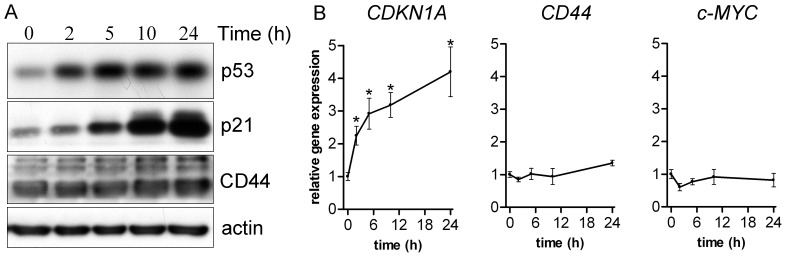
p53 protein does not repress CD44 expression in colon cancer cells. (A) Immunoblotting analysis of p53, p21 and CD44 protein levels in RKO colon cancer cells treated with ionizing radiation. Actin was used as loading control. (B) qRT-PCR results showing relative gene expression levels for *CDKN1*A (*p21*), *CD44,* and *c-MYC*. Data represent mean ± SEM of duplicate experiments;.(*, *P*<0.05 compared with t = 0).

The above findings do not exclude the possibility that wild-type p53 may (partially) suppress CD44 expression in normal and neoplastic intestinal epithelium upon activation by genotoxic stress. To address this possibility, we determined the effects of DNA damage-induced p53 activation on CD44 levels in human RKO colon cancer cells. These cells express wild-type p53 and K-Ras, and are diploid [26]. Of particular interest, RKO cells also contain wild type *APC* and *CTNNB1* genes and lack constitutive β-catenin/TCF-4-mediated transcription [27]. This is of importance since the transcriptional regulation of *CD44* by p53 might be masked by constitutive Wnt pathway activation, leading to β-catenin/TCF-4-mediated *CD44* expression. RKO cells were exposed to 10 Gy of γ-radiation after which expression of *CDKN1A* (*p21)* and *CD44* and were analysed by real-time qRT-PCR. Expression of *c-MYC*, a direct Wnt target gene [28], [29] was also assayed to control for the maintenance of a steady state of β-catenin/TCF-4-driven transcriptional activity. In addition, p53, p21, and CD44 protein levels were analysed by immunoblotting. As expected, ionizing radiation-induced DNA damage resulted in p53 stabilization (Figure 3A) and the consequent transactivation of p21 was observed at all time points (Figure 3A and B). However, *CD44* gene expression and CD44 protein levels did not decrease over time (Figure 3A and B), while *c-MYC* mRNA levels remained stable (Figure 3B). These data indicate that p53 is unable to repress *CD44* expression in human colon cancer cells.

**Figure 4:**
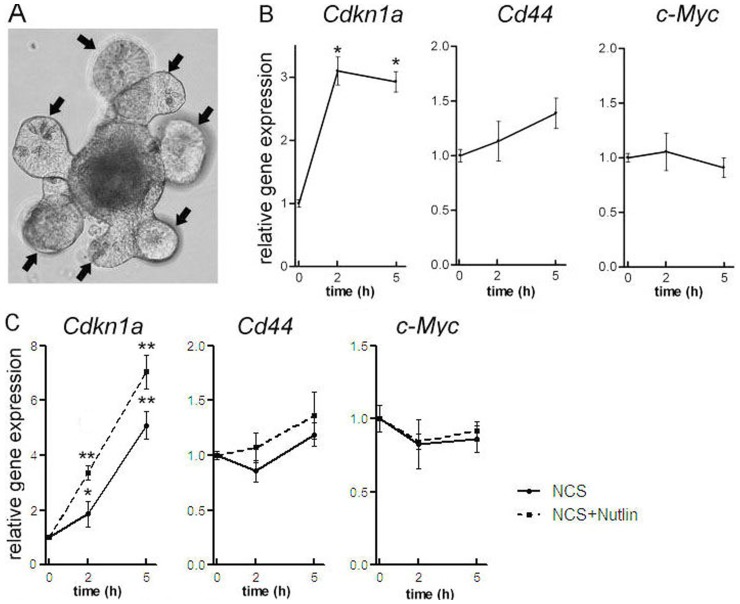
p53 is unable to repress *CD44* in the normal intestinal mucosa. (A) Crypt-villus organoid after one week of culture. Arrows indicate crypt-like compartments (B) qRT-PCR results showing relative gene expression levels after radiation treatment for *Cdkn1a*, *Cd44,* and *c-Myc.* Data represent mean ± SEM of duplicate experiments; (*, *P*<0.05 compared with t = 0). (C) qRT-PCR results showing relative gene expression levels after treatment with NCS alone or NCS plus nutlin for *Cdkn1a*, *Cd44* and *c-Myc* (*, *P*<0.05, **, *P*<0.01 compared with t = 0).

To extend our observations to normal intestinal epithelium, we next investigated the CD44 response to p53 activation in epithelial cells lining the crypt-villus axis of mouse small intestines. For this purpose, we employed *in vitro* cultured mouse intestinal epithelial crypts-villus organoids [19]. Organoids comprising multiple crypt domains (Figure 4A) were exposed to 10 Gy of γ-radiation after which *Cdkn1a*, *Cd44*, and *c-Myc* mRNA expression levels were analysed by real-time qRT-PCR. Similar to RKO cells, *Cdkn1a* mRNA levels were increased in the organoids in response to ionizing radiation (Figure 4B). These results are consistent with previous studies on radiation-induced p53 activation in the mouse crypt compartment [30]. *Cd44* mRNA expression was not significantly changed after radiation exposure, while expression levels *c-Myc* remained stable (Figure 4B). Similarly, chemical induction of p53 activation using NCS also resulted in increased levels of *Cdkn1a* mRNA. Simultaneous incubation with the p53 stabilizing agent nutlin further elevated *Cdkn1a* mRNA levels. In both conditions *Cd44* mRNA expression was not significantly altered, while *c-Myc* expression levels remained stable (Figure 4C).These results confirm our findings in the human RKO cells and in primary colon carcinomas, and demonstrate that *CD44* gene expression is not regulated by p53 in both normal and transformed intestinal epithelial cells.

## Discussion

The identification of p53 as a transcriptional repressor of CD44 expression in breast cancer [16] prompted us to investigate the relation between *p53* mutational status and CD44 expression in colon cancer. We demonstrate that, other than in breast cancer, *CD44* mRNA and protein levels are not increased in colon carcinomas with loss of functional p53, compared to tumours without *p53* mutations (Figure 1C, 2B). In addition, *CD44* expression in both normal and neoplastic intestinal epithelium was not affected by chemical or radiation-mediated activation of p53, indicating that p53 does not function as a transcriptional repressor of *CD44* in intestinal epithelial cells.

The observed tissue specific difference between breast and colon in transcriptional regulation of *CD44* might be explained by the complexity of p53 function. At least two features of the p53 protein are required for its gene regulatory function: p53 needs to recognize and bind a specific DNA sequences in the promoter of the target gene and p53 must recruit several transcriptional co-regulators (reviewed by Laptenko and Prives [14]). The *CD44* promoter contains a non-canonical p53 binding sequence [16], however, multiple interactions with co-activators and co-repressors as well as with the components of the general transcriptional machinery dictate its ability to direct promoter activation [14]. For example, interactions with ASPP1, BRCA1 or PTEN, or the coordinated activity of both p63 and p73, have been identified as determinants that direct specific responses [14], [31]. Differences in the expression and activity of these co-regulators between breast and intestinal epithelium could therefore contribute to a divergent role for p53 in the transcriptional control of the *CD44* gene in breast and colon epithelium and cancer cells. In addition, p53 can undergo several types of post-translational modification, including phosphorylation, acetylation and ubiquitination [32], which can direct promoter selection [33]. Hence, p53 function depends on a complex and tight regulation, and cell-specific modifications or interactions may explain its inability to repress CD44 in intestinal epithelial cells. Our finding that CD44 expression in normal intestinal epithelium and colon carcinomas is independent of p53 expression and p53 mutational status is of significance for understanding the pathogenesis of CRC and may have important therapeutic implications. Aberrant CD44 expression is advantageous for the growth, survival, and dissemination of tumour cells [1]. In CRC these biological functions of CD44 extend beyond its ability to antagonize the pro-apoptotic and cytostatic functions of p53 [16], [34]. This may, at least partly, explain the limited role of p53 in modulating the immediate phenotype of newly formed intestinal adenomas [35]. Furthermore, several studies have demonstrated that CD44 is a robust marker with functional importance for colon cancer stem cells [10], [11], [36]–[38]. These cells are believed to be relatively resistant to therapy and responsible for tumour-propagation, which makes CD44 an attractive target for cancer stem cell directed treatment, independent of p53.
